# Editorial: The interaction between viruses and the host immune system in persistent infection from a metabolic perspective

**DOI:** 10.3389/fcimb.2023.1300564

**Published:** 2023-11-22

**Authors:** Wei Xiong, Yong Feng, Yang Qiu, Yuncong Yuan, Chao Shen

**Affiliations:** ^1^ Lentigen Technology, Inc., a Miltenyi Biotec Company, Gaithersburg, MD, United States; ^2^ Department of Medical Microbiology, School of Basic Medical Sciences, Wuhan University, Wuhan, China; ^3^ State Key Laboratory of Virology, Wuhan Institute of Virology, Chinese Academy of Sciences, Wuhan, China; ^4^ Hubei Key Laboratory of Cell Homeostasis, College of Life Sciences, Wuhan University, Wuhan, China

**Keywords:** viruses, host immune system, interaction, persistent infection, metabolic

In recent years, our understanding of viral infections and their intricate dance with the host immune system has evolved significantly. The traditional view of host-pathogen interactions often centered around immune responses and the virus’s genetic makeup. However, emerging research has shed light on the crucial role of metabolism in determining the outcome of viral infections. The advancements in the field of immunometabolism have unveiled a fascinating dimension to this age-old battle. Viral persistence, a hallmark of certain infections, has been found to be profoundly influenced by the metabolic milieu within the host. The collections of articles and reviews set the stage for a deeper exploration of this captivating subject, highlighting the key objectives and the broader context of our research endeavor, proposing novel diagnostic, and therapeutic avenues for viral infection, and refining the design strategy of vaccines.

Though assessing the levels of Top1 expression in mitochondria of CD4 T cells from patients with chronic HCV or HIV infection, Dang et al. shed light on the molecular mechanisms underlying immune dysregulation during chronic viral infections and suggest that Top1mt inhibition may contribute to T cell dysfunction and mitochondrial damage. Restoring the Top1mt function could potentially be a therapeutic strategy for addressing these issues in individuals with chronic viral infections.

Zou et al. suggest that co-infection with HIV and TB significantly impacts the balance and function of γδ T cell subsets. The specific changes observed in subset percentages and cytokine production patterns highlight the complexity of the immune response in individuals with HIV/TB co-infection. Understanding these alterations in γδ T cell function in the co-infection context may provide valuable insights into the mechanisms underlying immune responses against HIV and/or MTB infections. Further research is warranted to delve deeper into these complexities and their implications for the development of therapeutic interventions.

Zhang et al. underscore the challenges in diagnosing and treating viral sepsis due to its underdiagnosis and heterogeneity. This heterogeneity poses significant difficulties in clinical management. The role of viral infections in disrupting glucose metabolism within infected cells has been established, and while this phenomenon is not yet fully understood in immune cells, it likely plays a crucial role in shaping antiviral immune responses. Though summarized the viral Infections and glucose Metabolism and challenges posed by viruses, especially SARS-CoV-2, demonstrate immune cell glucose metabolism from a viral perspective holds promise for advancing our understanding of the intricate interactions between viruses and the host immune system. Such insights are valuable for understanding viral infection pathogenesis and developing innovative vaccine and therapy strategies.

Zhong et al. aimed to compare the clinical characteristics of patients younger than 65 years old infected with the 2009 pandemic influenza A (H1N1) and the SARS-CoV-2 BA.2 variant (Omicron) to enhance the identification of these diseases and improve responses to the ongoing epidemic. Data from 127 patients with H1N1 diagnosed in 2009 and 3,265 patients with Omicron diagnosed in 2022 were collected. After matching for age, sex, and underlying diseases, data from 115 H1N1 patients (H1N1 group) and 230 Omicron patients (Omicron group) were analyzed. The study compared clinical manifestations between the two groups, identified possible independent risk factors using logistic regression, and analyzed factors predicting the time for nucleic acid negativization (NAN) using multiple linear regression. It highlights significant clinical differences between H1N1 and Omicron infections in patients under 65 years old. These differences include varying symptoms, laboratory findings, and treatment approaches. The study also emphasizes the longer duration of viral shedding in Omicron-infected patients. Understanding these distinctions is essential for accurate diagnosis and tailored management, especially in the context of the ongoing epidemic.

In summary, the Research Topic represents some significant advancements in the fields of immunology, metabolism, and virology. These findings hold great promise for enhancing our understanding of infectious diseases and may serve as crucial tools for diagnosing, preventing, and treating these illnesses.

## Author contributions

WX: Writing – original draft, Writing – review & editing. YF: Investigation, Methodology, Project administration, Resources, Supervision, Validation, Visualization, Writing – review & editing. YQ: Conceptualization, Data curation, Formal Analysis, Methodology, Software, Supervision, Validation, Visualization, Writing – review & editing. YY: Software, Writing – original draft, Writing – review & editing. CS: Conceptualization, Data curation, Funding acquisition, Investigation, Methodology, Project administration, Writing – original draft, Writing – review & editing.

